# A Chromosome-Level Genome Assembly of Yellowtail Kingfish (*Seriola lalandi*)

**DOI:** 10.3389/fgene.2021.825742

**Published:** 2022-01-19

**Authors:** Shuo Li, Kaiqiang Liu, Aijun Cui, Xiancai Hao, Bin Wang, Hong-Yan Wang, Yan Jiang, Qian Wang, Bo Feng, Yongjiang Xu, Changwei Shao, Xuezhou Liu

**Affiliations:** ^1^ Key Laboratory of Sustainable Development of Marine Fisheries, Ministry of Agriculture and Rural Affairs, Yellow Sea Fisheries Research Institute, Chinese Academy of Fishery Sciences, Qingdao, China; ^2^ Laboratory for Marine Fisheries Science and Food Production Processes, Qingdao National Laboratory for Marine Science and Technology, Qingdao, China; ^3^ China State Key Laboratory for Managing Biotic and Chemical Threats to the Quality and Safety of Agroproducts, Ningbo University, Ningbo, China

**Keywords:** *Seriola lalandi*, genome, adaptation, rapid growth, aquaculture

## Abstract

Yellowtail kingfish (*Seriola lalandi*) is a pelagic marine piscivore with a circumglobal distribution. It is particularly suitable for open ocean aquaculture owing to its large body size, fast swimming, rapid growth, and high economic value. A high-precision genome is of great significance for future genetic breeding research and large-scale aquaculture in the open ocean. PacBio, Illumina, and Hi-C data were combined to assemble chromosome-level reference genome with the size of 648.34 Mb (contig N50: 28.52 Mb). 175 contigs was anchored onto 24 chromosomes with lengths ranging from 12.28 to 34.59 Mb, and 99.79% of the whole genome sequence was covered. The BUSCOs of genome and gene were 94.20 and 95.70%, respectively. Gene families associated with adaptive behaviors, such as olfactory receptors and HSP70 gene families, expanded in the genome of *S. lalandi*. An analysis of selection pressure revealed 652 fast-evolving genes, among which *mkxb*, *popdc2*, *dlx6*, and *ifitm5* may be related to rapid growth traits. The data generated in this study provide a valuable resource for understanding the genetic basis of *S. lalandi* traits.

## Introduction

To develop environmentally friendly and economically sustainable aquaculture, it is necessary to understand the genetic basis of traits that currently limit/enhance development of domestic aquaculture (Rondeau et al., 2013). Genetic resources have been developed and widely used in agriculture and animal husbandry for decades, but only recently have they been used in selected aquaculture species (Ozaki et al., 2013; Dunham et al., 2014). There is still limited information on genetic variation on commercially important traits (Peterson et al., 2020). The methods used to develop these resources offer the best possibilities for genetic improvement or culture practices (Sodeland et al., 2013). Third-Generation Sequencing (TGS) has improved this area of research through high quality assemblies and decreasing costs, and this has enabled development of genetic resources for a greater number of species (Huete-Pérez and Quezada, 2013; Lee et al., 2016; Lv et al., 2020).

Yellowtail kingfish (*Seriola lalandi*) is an excellent marine economic fish. It has a number of beneficial traits for open ocean aquaculture systems, including large body size, rapid growth, and high-quality flesh (Orellana et al., 2014; Sanchís-Benlloch et al., 2017). Similar in taste to tuna or mackerel, yellowtail kingfish have a large market worldwide and are a popular fish used in sushi (Purcell et al., 2015). These make them a good candidate for aquaculture. Since the 1990s, extensive research in Japan has focused on artificial breeding and breeding technology for *S. lalandi* (Sano, 1998). In China, aquaculture of *S. lalandi* began in 2001 (Jiang et al., 2001), along with biological research, including studies of embryogenesis, seedling cultivation, and effects of salinity stress on growth (Shi et al., 2019; Xu et al., 2019; Liu A et al., 2021).

Here, we report a chromosomal-level genome assembly of *S. lalandi*. Our evolutionary and comparative genomic analysis provide insights into the adaptability of the species to the external environment. Furthermore, the genome analysis provide a valuable resource for further studies of the genetic basis of traits of *S. lalandi*.

### Value of the Data

This is the first chromosomal-level genome assembly in *Seriola* genus*.* It could be a valuable resource to conduct a comparative analysis among the species in the genome of the *Seriola* genus and for further studies of the genetic basis of traits of *S. lalandi.*

## Materials and Methods

### Sampling and Sequencing

Yellowtail kingfish specimens were collected from Dalian Futai Marine Products Farming Co., Ltd. (Dalian, China). Total genomic DNA of a male fish muscle sample was extracted using the QIAamp DNA Mini Kit (QIAGEN, Hilden, Germany) following the manufacturer’s protocols. We constructed two paired-end libraries (insert sizes of 200 and 500 bp) following the manufacturer’s protocol (Chromium Genome v1, PN120229). The libraries were sequenced on the BGISEQ-500 platform to obtain PE 2 × 150 bp reads. The extracted DNAs were also used to construct a 20 kb library following the PacBio protocol (Pacific Biosciences, Menlo Park, CA, United States). The libraries were then sequenced on the PacBio Sequel platform. We obtained 48.74 and 106.76 Gb of raw sequence data using the BGISEQ-500 and PacBio platforms, respectively (Supplementary Table 1).

To construct chromosome-level assemblies, the Hi-C technique was used. A Hi-C library was prepared following the strategy described by Rao et al. (Rao et al., 2014) using blood samples with an ∼300 bp insert size. Using the BGISEQ-500 platform to sequence the Hi-C library, we obtained 87.60 Gb of raw Hi-C data (Supplementary Table 1).

Four tissues (brain, pituitary, liver, and muscle) were collected for RNA sequencing. RNA from each tissue was extracted and treated with DNase I (TAKARA, Kusatsu, Japan) to remove genomic DNA. For each tissue, a paired-end RNA-sequencing library was constructed with an insert size of 300 bp and then sequenced on the Illumina HiSeq 2,500 platform to generate PE 2 × 150 bp. One muscle specimen was also used to construct an Iso-Seq library and then sequenced on the PacBio Sequel platform. In total, we obtained 307.14 and 26.89 Gb of raw sequence data using the Illumina HiSeq 2,500 and PacBio platforms, respectively (Supplementary Table 1).

### Genome Assembly, Chromosome Anchoring, and Genome Annotation

Before genome assembly, we estimated the genome by a k-mer analysis using Jellyfish v2.2.6 (Marçais and Kingsford, 2011). For this, a series of k-mers (17, 19, and 21) were extracted from the BGISEQ-500 sequencing data and the frequency of each kmer was calculated. The heterozygosity rate was estimated using 17-mers using GenomeScope v2.0.0 (Supplementary Figure 1). Considering the C-value (0.7) from the Animal Genome Size Database, the estimated genome size of *S. lalandi* was 684.60 Mb.

Canu v1.8 was used for the self-correction of long reads sequenced with the PacBio Sequel platform. Then, the corrected reads were assembled using wtdbg2 v2.5 (options: -x rs -g 750 m) (Ruan and Li, 2020). Pilon v1.23 (Walker et al., 2014) was used to polish contigs with short reads by three rounds of alignment. The Hi-C short reads were aligned to the scafiolds using Juicer (Durand et al., 2016) and anchoring was performed using 3D-DNA v180419 (Dudchenko et al., 2017). We finally used Juicebox Assembly Tools v1.9.9 (Durand et al., 2016) to correct the connections. The completeness of the final assembly was assessed using BUSCO v.4.0 (Simão et al., 2015).

Both homology-based and *de novo* predictions were used to annotate repetitive sequences. Transposable elements were identified using RepeatMasker v4.0.7 (http://www.repeatmasker.org) and RepeatProteinMask v1.36 with Repbase v17.01 (Bao et al., 2015). A *de novo* transposable element library was constructed using RepeatModeler v1.0.11 (http://www. repeatmasker.org/RepeatModeler.html) and was then used to predict repeats using RepeatMasker.

To annotate gene structures, we used homology-based prediction, transcriptome-based prediction, and *de novo* prediction. For homology-based annotation, the protein sequences of eight teleost species downloaded from NCBI, including *Seriola lalandi dorsalis*, *Seriola dumerili, Seriola quinqueradiata*, *Seriola rivoliana*, *Echeneis naucrates*, *Oryzias latipes*, *Danio rerio*, and *Takifugu rubripes*, were aligned to the genome assembly by BLAT v3.6 (Kent, 2002), and then GENEWISE v2.4.0 (Birney et al., 2004) was used to predict gene structures. For next-generation RNA-sequencing annotation, data were aligned to the genome assembly using HISAT2 v2.1.0 (Kim et al., 2015) and the alignments were fed to StringTie v1.3.5 (Pertea et al., 2015) to assemble the transcriptome. TransDecoder v5.0.2 (https://github.com/TransDecoder/TransDecoder/) was used to predicate ORFs and identify candidate gene structures. For third-generation RNA-sequencing annotation, long-read RNA-seq (PacBio Iso-Seq) transcripts were obtained by removing the redundant sequences using cd-hit-est v4.8.1 (Li and Godzik, 2006). Then, the non-redundant transcripts were mapped to the genome by BLAT and assembled using PASA v2.0.2 (https://github.com/PASApipeline/PASApipeline/). For *de novo* prediction, the gene structures were analyzed on the repeat-masked genome assembly using AUGUSTUS v2.5.5 (Stanke et al., 2006), GlimmerHMM v3.0.4 (Allen et al., 2006), and GENSCAN (Burge and Karlin, 1998). Finally, genes predicted from the above methods were merged to obtain a consensus gene set using Evidence Modeler (EVM). For the functional annotation of the gene sets, the protein sequences of these genes were aligned against sequences in public protein databases, including, NR, KEGG, SwissProt, GO, InterPro, and Trembl, to identify homologues using Blastp v2.2.26 with an E-value cutoff of 1e-5.

### Phylogenetic Analysis and Gene Family Expansion

To determine single-copy genes of *S. lalandi* and other species (*S. dumerili*, *S. quinqueradiata*, *S. rivoliana*, *E. naucrates*, *O. latipes*, *D. rerio*, *T. rubripes*, *Larimichthys crocea*, *Oreochromis niloticus*, and *Caranx melampygus*), the TreeFam pipeline (Li et al., 2006) was used. Before generating the alignment, the longest transcript of each gene was selected and protein sequences shorter than 50 amino acids were filtered out. Then, Blastp searches were performed for all protein sequences with an E-value cut-off of 1e-5, and fragmented alignments were merged using SOLAR. Hcluster was used to filter segments, group genes, and determine single-copy orthologue families. The phylogenetic tree was inferred using multiple alignments from the single-copy genes using RaxML-ng v0.9.0 (Kozlov et al., 2019) under the site-heterogeneous GTR + G4 model with maximum likelihood estimation (ML).

An ultrametric tree was inferred using r8s v1.71 with fossil records from the TimeTree website (http://www.timetree.org) for calibration. An MCMCTREE analysis implemented in PAML v4.5 (Yang, 1997) was employed to estimate divergence times. CAFÉ v5.0 (De Bie et al., 2006) was used to assess gene family size dynamics, and families with *p* < 0.05 showed significant expansion or contraction. GO and KEGG pathway enrichment analysis were used to analyze the expanded and contracted genes.

### Positive Selection Analysis

To identify positively selected genes (PSGs), we re-determined single-copy orthologues shared among five species (*E. naucrates*, *T. rubripes*, *O. latipes*, *D. rerio,* and *S. lalandi*) and constructed a phylogenetic tree. Based on the new phylogenetic tree and single-copy genes, we estimated the rate ratio (*ω*) of non-synonymous to synonymous nucleotide substitutions using CodeML (PAML package) to examine selective constraint. After obtaining high-quality alignments using prank v.100802 (Löytynoja and Goldman, 2010), Gblocks v0.91b (Castresana, 2000) was used to eliminate poorly aligned positions and divergent regions. Finally, the signature of positive selection (*d*_*N*_ */d*_*S*_ > 1) was identified using the PAML branch site model. GO and KEGG pathway enrichment analysis were used to evaluate PSGs.

## Results and Discussion

### Genome Features

**FIGURE 1:**
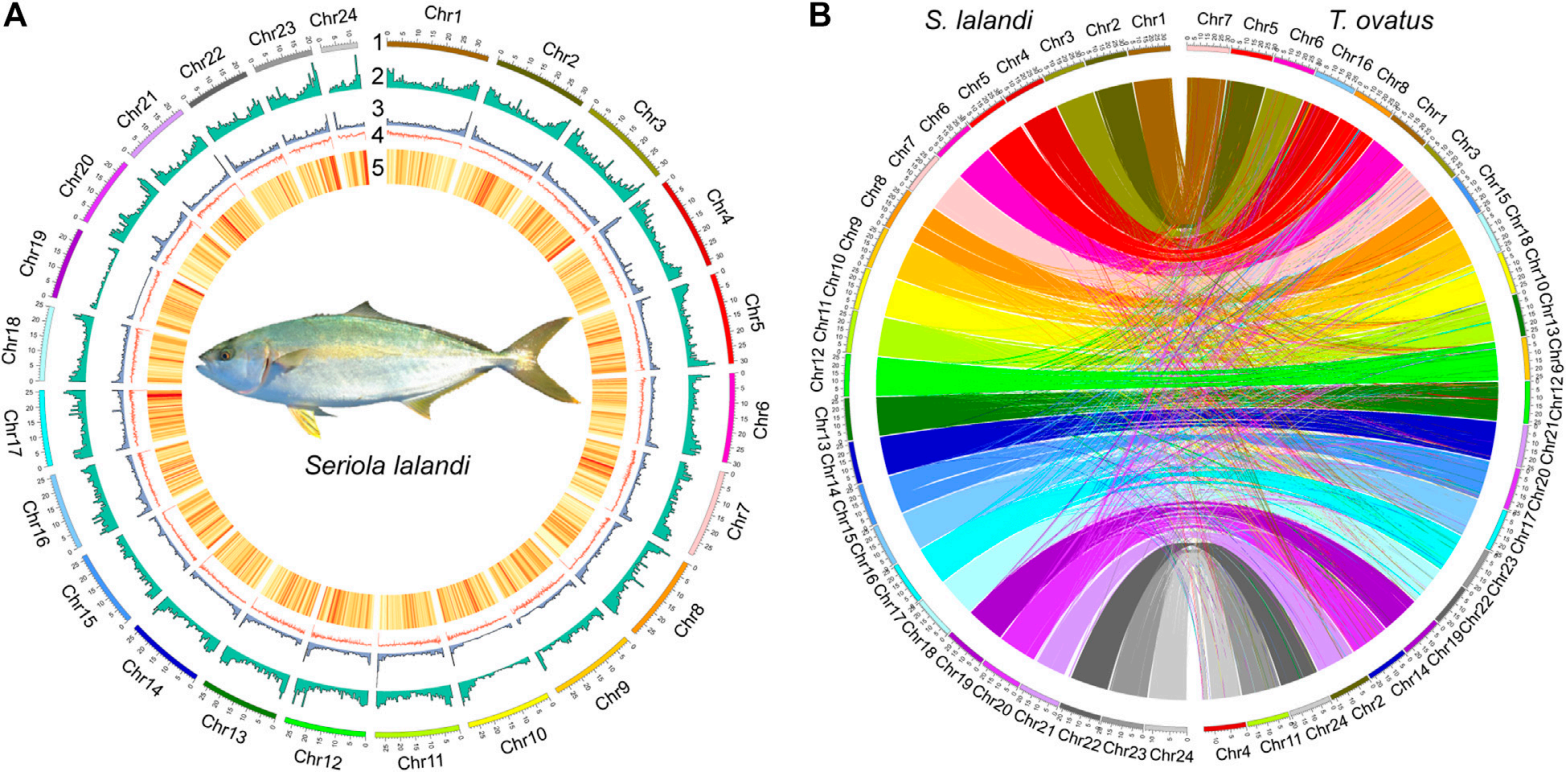
Genome assembly and comparison. **(A)** Circos graph of genome statistics. Genomic features. From outer to inner circles: 1, represents chromosomes; 2, distribution of DNA transposons; 3, distribution of retrotransposons; 4, GC content; 5, gene distribution density; 6, each line joins paralogous genes at different chromosomes. 2–5 are drawn with 500 Kb sliding windows. **(B)** Genome comparison between *S. lalandi* and *T. ovatus*. The *S. lalandi* chromosomes are on the left, and the *T. ovatus* chromosomes are on the left.

To generate a high-quality reference genome, we combined PacBio, Illumina, and Hi-C data (Supplementary Table 1). PacBio CLRs with coverage of 165 × were used for genome assembly. The draft assembly was 648.34 Mb, with 277 contigs, a contig N50 of 28.52 Mb, and a GC content of 40.79% (Supplementary Table 2). Using ∼52 Gb (∼87×) of valid Hi-C data, we anchored 175 contigs onto 24 chromosomes (Figure 1A, Supplementary Figure 2) (Shi et al., 2017). The lengths of the 24 chromosomes ranged from 12.28 to 34.59 Mb, and 99.79% of the whole genome sequence was covered (Supplementary Table 3). To evaluate the completeness of the assembly, the BUSCO database (actinopterygii_odb10) and RNA-seq data were used. The genome contained 94.20% complete BUSCOs and the average mapping rate of transcriptome data was 96.30% (Supplementary Table 4). The published *Trachinotus ovatus* chromosome-level genome was used to validate the accuracy of the assembly of the chromosomes (Zhang et al., 2019); 567.01 MB synteny blocks (each synteny block > 500 bp) were consistent with the assembled chromosomes (Figure 1B).

Repetitive elements comprised 22.46% of the *S. lalandi* genome, similar to the estimate in the *T. ovatus* genome (20.25%, 655 Mb) (Zhang et al., 2019). The most abundant transposable elements (TEs) were DNA transposons (11.51%), followed by long terminal repeats (LTRs, 4.93%) and long interspersed elements (LINEs, 3.85%) (Supplementary Figure 4). We integrated *de novo*, homology-based and transcriptome-based methods to predict a protein-coding gene set comprising 22,674 genes (Supplementary Table 5), and which 20,568 (90.71%) matched entries in a public database (Supplementary Table 6). We identified 95.70% complete BUSCOs from 22,674 protein-coding genes.

### Phylogenetic Relationships and Genomic Comparison

**FIGURE 2:**
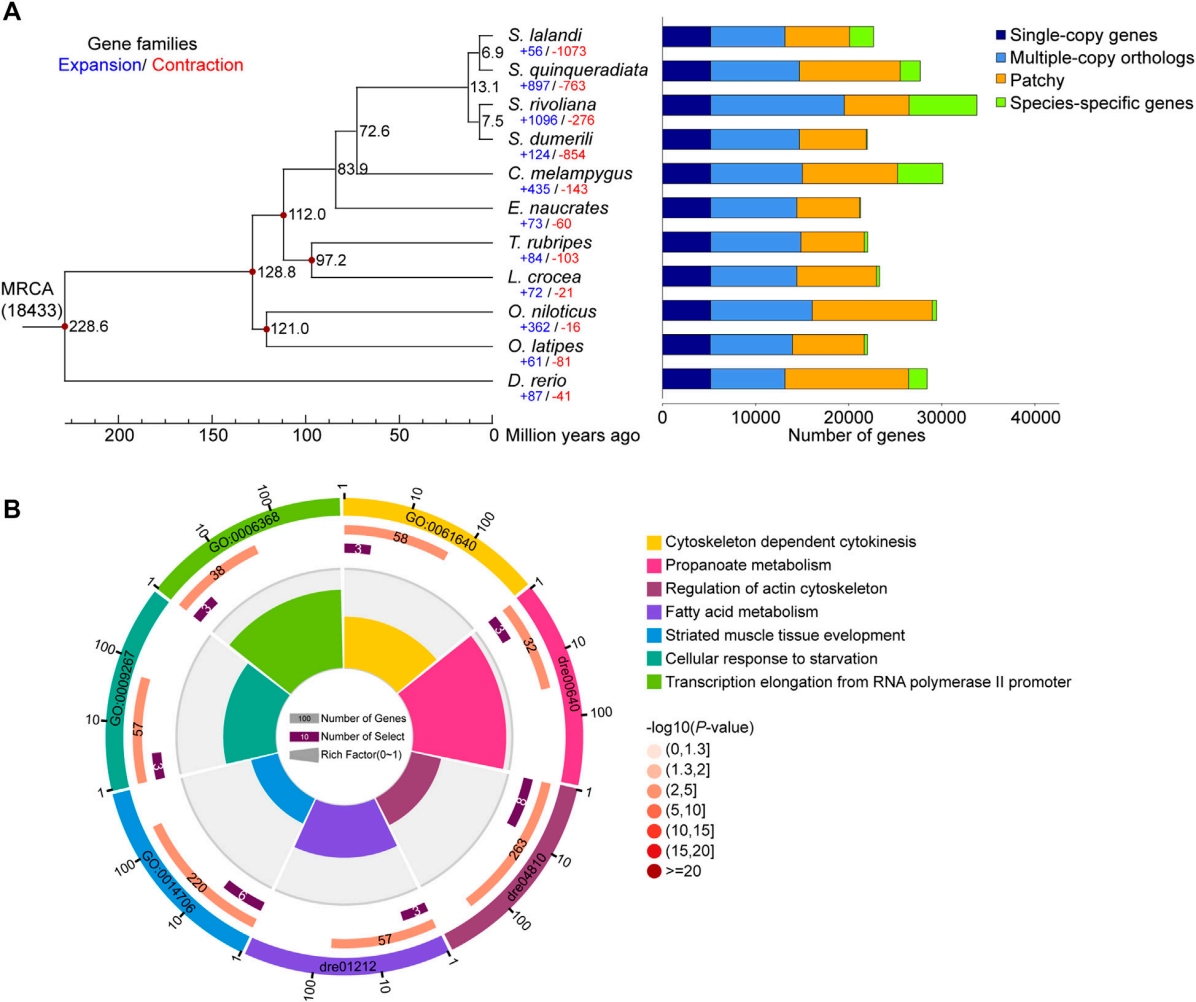
Genome evolution analysis. **(A)** Phylogenetic tree of 11 teleost genomes, which was constructed using 5,067 single copy orthologous genes. The black numbers on the branches indicate the estimated diverge times in millions of years ago, and the blue and red numbers represent the expanded and contracted gene families. The different types of orthologous relationships are shown on the right. **(B)** The enrichment analysis of 148 positively selected genes detected in *S. lalandi* genome.

We constructed a phylogenetic tree of *S. lalandi* and 10 teleost fish (*S. dumerili*, *S. quinqueradiata*, *S. rivoliana*, *E. naucrates*, *O. latipes*, *D. rerio*, *T. rubripes*, *L. crocea*, *O. niloticus*, and *C. melampygus*) based on 5,067 single-copy genes (Figure 2A, Supplementary Table 7). According to the phylogeny and the fossil record of teleosts, we dated the divergence of *Seriola* from the other teleost species to approximately 72.6 million years ago (Figure 2A).

We detected 56 significantly expanded and 1,073 significantly contracted gene families (*p* < 0.05) in *S. lalandi* (Figure 2A). Compared with teleost fish except of *Seriola*, the HSP70 family with 19 HSP70 genes was expanded (Supplementary Table 8). We found five *hspa12* genes, including *hspa12a*, *hspa12b*, *hspa12l-1*, *hspa12l-2*, and *hspa12l-3*, which was more than observed in *E. naucrates* (2), *O. latipes* (2), *D. rerio* (2), *T. rubripes* (2), *C. melampygus* (3), and *L. crocea* (3) (Liu et al., 2019). In *S. lalandi*, there were three *hspa12l* gene copies. HSP70 is a well-known stress protein (Clark and Peck, 2009), and the expansion of the HSP70 family in *S. lalandi* may contribute to its adaptation to changes in the aquatic environment.

Yellowtail kingfish is a migratory marine fish with high olfactory sensitivity (Martínez-Montaño et al., 2016). We identified 147 olfactory receptor (OR)-like genes from the *S. lalandi* genome, including subfamily "Delta" (68), "Eta" (49), "Zeta" (12), "Epsilon" (9), "Beta" (6), "Thet" (2), and "Kappa" (1) (Supplementary Table 9). The expanded subfamilies "Delta" and "Epsilon" are important for the perception of water-soluble odorants (Cong et al., 2019). Most teleosts possess one or two “Beta” OR genes, which are important for detecting both water-soluble and airborne odorants (Liu H et al., 2021). However, subfamily "Beta" of olfactory receptor was expanded in *S. lalandi*. These expansions may contribute to the olfactory detection ability of the species, which could be useful for feeding and migration (Bett and Hinch, 2016).

### Fast-Evolving Genes in Yellowtail Kingfish

PSGs are often associated with adaptive evolution and may contribute to new or improved functions. To understand the selective pressure operating on *S. lalandi*, we compared the orthologues of five teleost species (*E. naucrates*, *T. rubripes*, *O. latipes*, *D. rerio*, and *S. lalandi*) and identified 652 fast-evolving genes, including 148 PSGs (*d*_*N*_ */d*_*S*_ > 1) and 504 genes that contain positively selected sites in *S. lalandi* (Supplementary Table 10). Consistent with the large body size and fast swimming ability, an enrichment analysis revealed that the PSGs were involved in striated muscle tissue development (GO:0014706), regulation of actin cytoskeleton (dre04810), and fatty acid metabolism (dre01212) (Figure 2B).

Muscle tissue development is associated with the growth rate, which is a major economic trait in animal production. Several genes involved in muscle tissue development (*klf2a*, *klhl41b*, *cdk9*, *ndrg4*, *mkxb*, and *popdc2*) showed rapid evolution in *S. lalandi* and likely contribute to the rapid growth of the species (Supplementary Table 10). Fast growing muscles also require increased bone support. Two genes, *dlx6* and *ifitm5,* were involved in skeletal system development and promote bone formation to support the large body (Supplementary Table 10). Based on the strong muscle and skeletal systems, muscle contraction-related genes (*arhgef12b*, *ramp2*, *tnnt2a*, *tnni1a*, *cald1a*, and *tnnt2a*) with positively selected sites may provide support for fast swimming (Supplementary Table 10). Furthermore, fatty acid metabolism-related fast-evolving genes (*hsd17b12b*, *acadm*, *mecr*, *lipg*, and *hao1*) also contributed to energy consumption and growth (Supplementary Table 10). Moreover, some genes with positively selected sites were associated with growth (*ficn*, *tgfbr3*, *igf1ra*, *gpc1b*, *rnf11*, and *tgfbrap1*) (Supplementary Table 10).

We identified other fast-evolving genes, such as the pheromone response gene *ora2*, ear development gene *ddt*, and sensory perception gene *ppef1*, with potential roles in the perception of the external environment (Supplementary Table 10). Fast-evolving genes involved in nervous system development and the regulation of neurotransmitter secretion (*rab33a*, *fstl5*, and *cplx2*) provided a tissue basis for sensitive sensory systems (Supplementary Table 10). The fast-evolving genes *ldha* and *rnf152* may contribute to adaptation to adverse environmental conditions, such as hypoxia and starvation (Supplementary Table 10).

## Conclusions

We sequenced and assembled the genome of *S. lalandi* using Illumina shotgun, PacBio SMRT, and Hi-C data, providing the first chromosome-level genome assembly for the genus *Seriola*. Basing on multiple annotation strategies, we obtained 22,674 protein-coding genes with minimal redundancy. Further genomic analysis revealed gene families associated with expansions of HSP70 and olfactory receptor gene families, and the rapid evolution of muscle and skeletal system development genes, providing insight into the genetic basis underlying the physiological characteristics of *S. lalandi* and its adaptability to the external environment. We believe these new resources will promote genetic research and accelerate the genetic breeding process for *S. lalandi*.

## Data Availability

The datasets presented in this study can be found in online repositories. The names of the repository/repositories and accession number(s) can be found below: GenBank, JAIQDC010000001.1-JAIQDC010000031.1; NCBI BioProject, PRJNA754209.

## Abbreviations

N50, median size; Gb, gigabase pairs; Mb, megabase pairs; kb, kilobase pairs; PacBio, Pacific Biosciences; SMRT, single molecule, real-time; BUSCOs, Benchmarking Universal Single-Copy Orthologs; GC, guanine-cytosine; GO, Gene Ontogeny; KEGG, Kyoto Encyclopedia of Genes and Genomes; Mb, megabase pairs; ML, maximum likelihood; MYA, million years ago; NCBI, national center for biotechnology information; HSP70, heat shock protein 70 family.
