# Supplementary material for: A Chromosome-Level Genome Assembly of Yellowtail Kingfish (Seriola lalandi)
Source: Front Genet. 2022 Jan 19;12:825742. doi: 10.3389/fgene.2021.825742 (PMC8807568; doi:10.3389/fgene.2021.825742)
Supplement: Supplementary file 3 [file Table15.docx]

**Supplementary Figure 1.** The k-mer distribution of Illumina paired-end reads.

**Supplementary Figure 2.** Hi-C chromosome contact map. Darker red of a block indicates higher contact intensity.

**Supplementary Figure 3.** Divergence of 4 types of transposon elements.

**Supplementary Table 1.** Data statistics of this study.

**Supplementary Table 2.** Comparisons of other published Seriola assemblies.

**Supplementary Table 3.** Genome chromosome statistics.

**Supplementary Table 4.** Summary of genome alignment based on the transcriptome data.

**Supplementary Table 5.** Statistics of the number of gene sets with different annotation methods.

**Supplementary Table 6.** Statistics of gene set function annotation.

**Supplementary Table 7.** Statistics of all gene families.

**Supplementary Table 8.** Statistics of HSP70 gene family.

**Supplementary Table 9.** Statistics of olfactory receptor (OR) gene family.

**Supplementary Table 10.** Statistics of 652 fast-evolving genes.
