# Supplementary figures and images for: A Chromosome-Level Genome Assembly of Yellowtail Kingfish (Seriola lalandi)
Source: Front Genet. 2022 Jan 19;12:825742. doi: 10.3389/fgene.2021.825742 (PMC8807568; doi:10.3389/fgene.2021.825742)

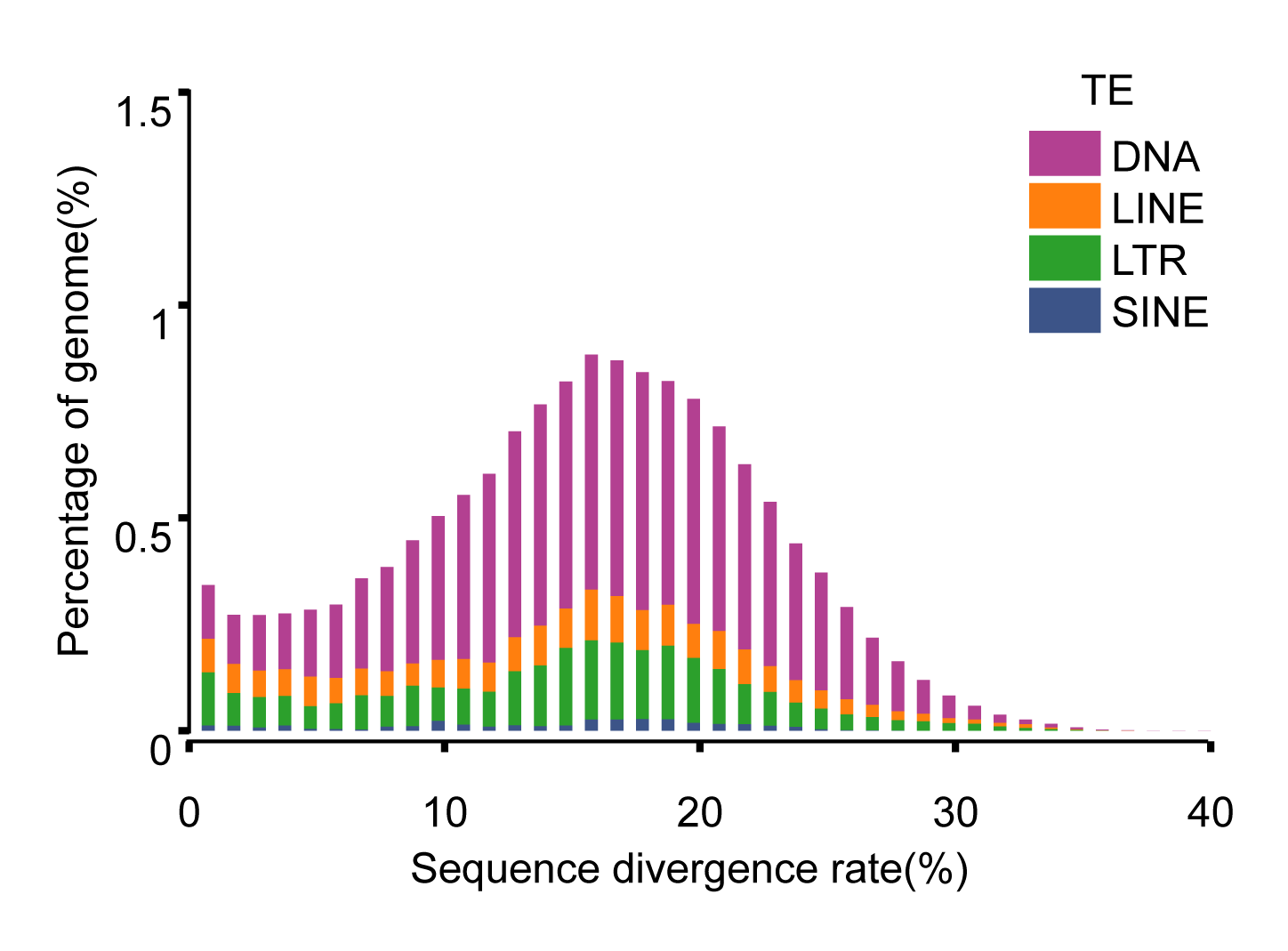

Supplement: Supplementary file 5 [file Image3.TIF]

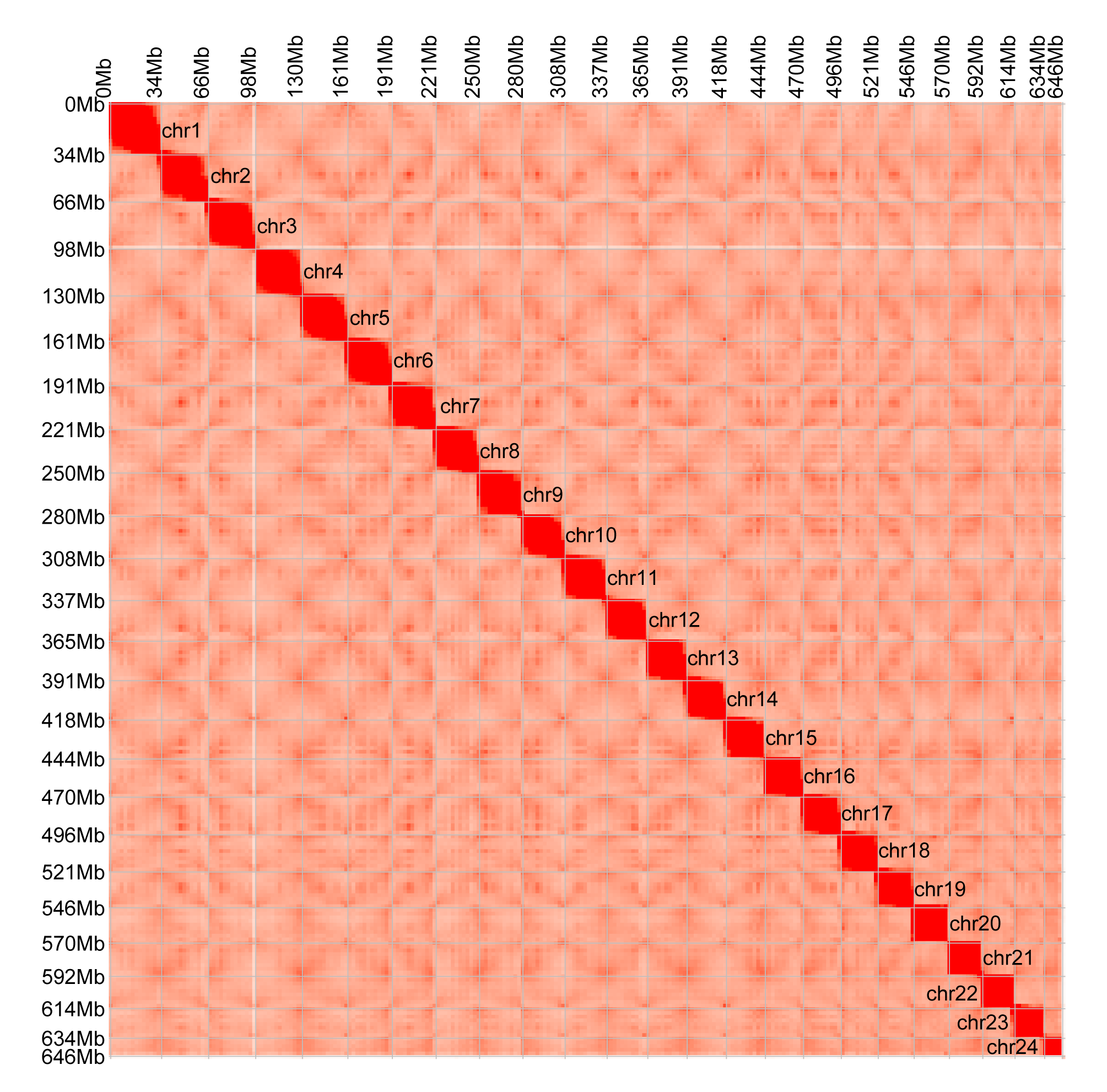

Supplement: Supplementary file 6 [file Image2.TIF]

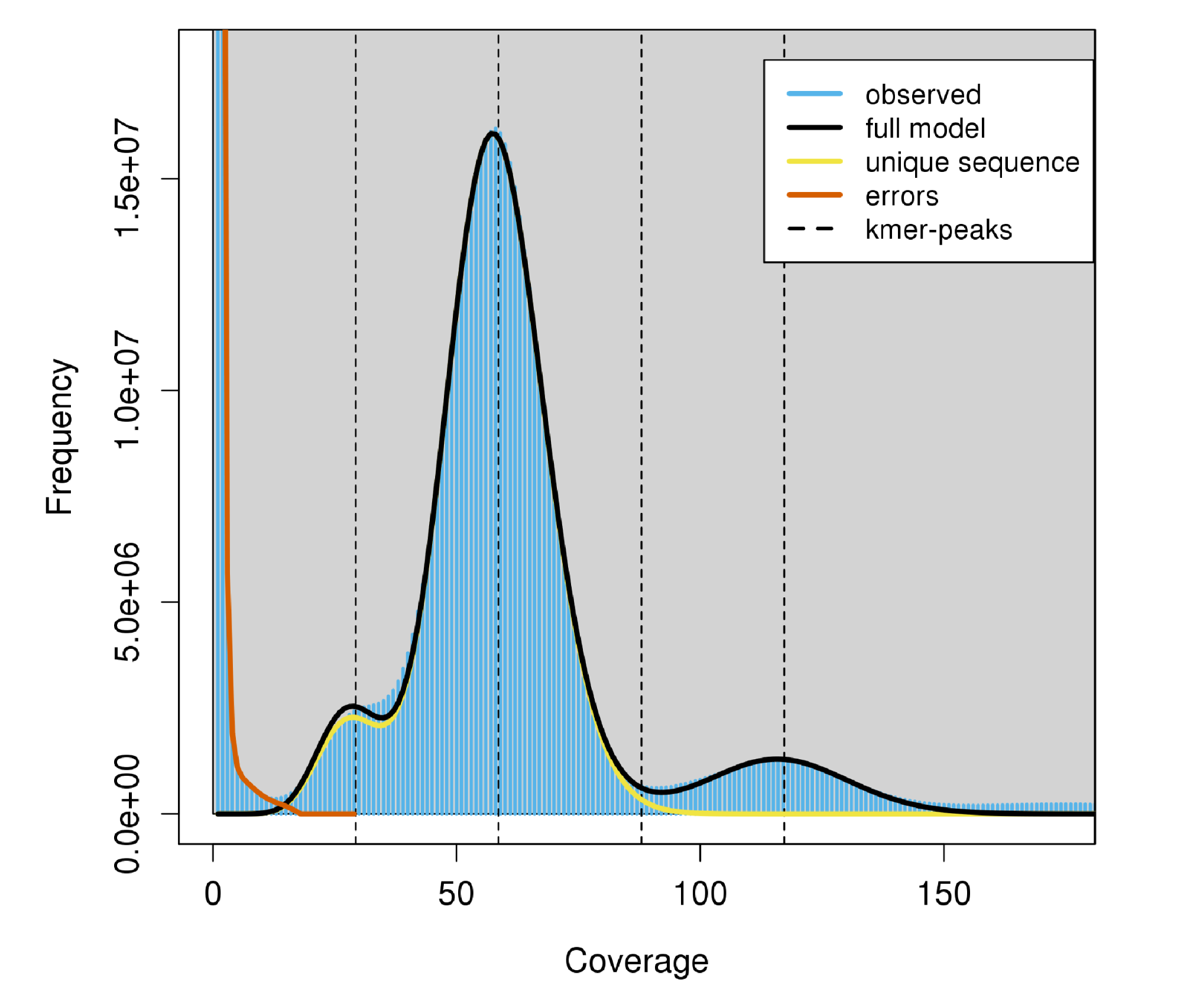

Supplement: Supplementary file 7 [file Image1.TIF]
